# Impact of evidence-based nursing interventions on psychological status and myocardial injury in patients with myocardial infarction following percutaneous coronary intervention for reperfusion injury

**DOI:** 10.3389/fphys.2025.1597416

**Published:** 2025-07-25

**Authors:** Ni Li, Xi Chen

**Affiliations:** ^1^ Department of Cardiology, West China Hospital, West China School of Nursing, Sichuan University, Chengdu, Sichuan, China; ^2^ Department of Critical Care Medicine, West China Hospital, West China School of Nursing, Sichuan University, Chengdu, Sichuan, China

**Keywords:** evidence-based nursing, myocardial infarction, percutaneous coronary intervention, reperfusion injury, myocardial injury, psychological status

## Abstract

**Objective:**

Percutaneous coronary intervention for myocardial infarction can cause reperfusion injury with both physical and psychological impacts on patients. This study aims to assess how evidence-based nursing affects psychological state and myocardial injury in reperfusion injury in patients with myocardial infarction following percutaneous coronary intervention.

**Method:**

Ninety patients with myocardial infarction who underwent percutaneous coronary intervention and were confirmed to have postoperative reperfusion injury were randomly divided into a control group that received conventional care or an intervention group that received evidence-based nursing intervention. The levels of cardiac function indicators (left ventricular ejection fraction and left ventricular end-diastolic diameter), myocardial injury markers (N-terminal pro-b-type natriuretic peptide and cardiac troponin I), psychological status (Self-Rating Anxiety Scale and Self-Rating Depression Scale scores), the incidence of complications, mortality rate, and satisfaction with nursing care were compared before and after the intervention.

**Results:**

Compared to pre-intervention, after the intervention, both groups showed reductions in left ventricular end-diastolic diameter, N-terminal pro-b-type natriuretic peptide, cardiac troponin I levels, Self-Rating Anxiety Scale and Self-Rating Depression Scale scores, with an increase in left ventricular ejection fraction. The intervention group demonstrated superior outcomes in all these metrics compared to the control group, with lower rates of complications and mortality, and higher satisfaction levels.

**Conclusion:**

Implementing evidence-based nursing in patients with myocardial infarction undergoing percutaneous coronary intervention for reperfusion injury can improve cardiac function, alleviate myocardial injury and psychological status, reduce the incidence of complications, and enhance patient satisfaction.

## Introduction

Myocardial infarction (MI), a major global health concern, is defined as the death of heart muscle tissue resulting from prolonged ischemia, often leading to severe complications (Zheng et al., 2021). Percutaneous coronary intervention (PCI) is an effective method for treating acute MI (Pu et al., 2024). However, PCI is frequently complicated by reperfusion injury, a counterintuitive phenomenon in which the return of blood flow to ischemic tissue triggers further cellular damage, increasing myocardial cell death and reducing the amount of salvageable myocardium (Zheng et al., 2021; Visker et al., 2024). This injury is driven by complex mechanisms, including oxidative stress, inflammatory responses, and ferroptosis, all of which can worsen myocardial damage and hinder recovery (Li et al., 2021). Reperfusion injury is a significant and crucial factor contributing to periprocedural MI. It has been demonstrated to be closely associated with an increased mortality rate and the occurrence of adverse outcomes after acute MI (Armillotta et al., 2025). From a psychological perspective, the occurrence of reperfusion injury after PCI often imposes substantial psychological stress on patients. They may experience anxiety, depression, and other negative emotions due to concerns about disease progression and treatment efficacy, which are detrimental to their recovery (Liu F. et al., 2022; Peng et al., 2025).

It has been reported that Evidence-based nursing (EBN) interventions have shown significant potential in alleviating psychological distress, such as anxiety and depression, while also improving cardiac outcomes in MI patients undergoing PCI (Gao et al., 2022). EBN is a process in which nurses carefully, wisely, and clearly integrate scientific research findings with clinical experience and patient preferences during the planning of nursing activities to obtain a basis for decision-making (Hou et al., 2024). The steps of EBN include formulating clinical questions, searching for external evidence, appraising the scientific quality, developing nursing plans by combining patient needs and clinical experience, and finally implementing nursing care (Machado et al., 2025). EBN represents a paradigm shift in contemporary healthcare, moving away from traditional, experience-driven practices toward a more scientifically grounded and patient-focused approach (Wang et al., 2020). Its application has proven particularly useful in managing complex psychological and physiological conditions, such as post-stroke depression, anxiety disorders, and perioperative care, where it has demonstrated significant improvements in patient wellbeing and recovery (Geng et al., 2021; He et al., 2021). In addition, combining EBN with exercise rehabilitation can improve cardiac function and physiological indicators, enhance treatment adherence and satisfaction, and reduce the incidence of postoperative complications in acute MI patients after PCI (Liu X. et al., 2022).

Therefore, this study aims to assess how EBN interventions affect psychological and physical consequences in MI patients with reperfusion injury after PCI, with the aim of providing scientific evidence for clinical nursing practice and improving patients’ prognosis and quality of life.

## Materials and methods

### Ethical approval

All experimental procedures were approved by the Medical Ethics Committee of West China Hospital/West China School of Nursing, Sichuan University. The patient has been notified about the information contained in the consent form.

### Study design

This study was conducted as a prospective, randomized, controlled, and assessor-blind trial. It aimed to explore the application of EBN intervention in reperfusion injury after PCI in patients with MI, as well as its impact on psychological state and myocardial injury. The experimental design comprised two groups, where each participant was randomly assigned to receive either conventional care (control group) or EBN intervention (intervention group) in a parallel-arm design.

### Participants

The patients who underwent PCI for MI were diagnosed with postoperative reperfusion injury at West China Hospital/West China School of Nursing, Sichuan University between August 2020 and August 2022.

Inclusion criteria: Patients whose MI diagnosis conformed to the relevant criteria in the “Integrated Traditional Chinese and Western Medicine Diagnosis and Treatment Guidelines for Acute Myocardial Infarction,” and who experienced persistent pain in the anterior chest or behind the sternum for at least 30 min; patients with elevated cardiac biomarkers; patients showing peaked T waves and upwardly convex ST segments on their electrocardiograms; patients with vascular stenosis of 70% or greater; patients who voluntarily enrolled in the study and signed informed consent forms.

Exclusion criteria: Patients with serious underlying conditions; patients with malignant tumors, mental illnesses, or poor cooperation in the study; patients with incomplete data.

### Randomization and masking procedures

Participants were allocated to either the intervention or control group in a 1:1 ratio through a computer-generated random number sequence. A randomizer tool was employed to produce a unique random allocation number for each participant. These generated random numbers were securely concealed or safeguarded. The sequence of random allocations remained undisclosed to both the participants and the research team until the group assignments were finalized. An independent researcher was responsible for carrying out the group assignments by cross-verifying the random allocation numbers and allocating participants to their corresponding groups. The individuals responsible for outcome assessment and statistical analysis of the data were kept unaware of the group allocations.

### Nursing methods

During their hospital stay, the control group received conventional care, which encompassed the following: Vital signs, such as blood pressure, heart rate, and respiratory rate, were continuously monitored to ensure stability. Electrocardiogram readings were closely observed to detect any changes in cardiac activity and promptly report abnormalities. For patients requiring postoperative anticoagulant therapy, nurses were trained to understand the proper use and precautions of each medication, and this information was effectively communicated to patients. For those on other medications (e.g., beta-blockers, angiotensin receptor blockers), nurses closely monitored treatment efficacy and potential side effects. In addition, vascular access was maintained to ensure patency and prevent infections or damage to the surgical site. The surgical site was regularly checked for signs of bruising, hematoma, or bleeding, and any issues were addressed immediately. Meanwhile, individualized rehabilitation plans were created, including gradual increases in physical activity and cardiac rehabilitation, while ensuring patients did not overexert themselves. Patients were also guided through rehabilitation exercises to improve cardiopulmonary function. Psychological concerns were addressed by offering timely counseling to boost patients’ confidence in the recovery, and open communication was maintained with families to enhance home care practices. Guidance on daily living was provided, such as advising patients and their families on the importance of a low-fat, low-salt, and low-cholesterol diet, avoiding spicy or irritating foods, maintaining a regular sleep schedule, ensuring good indoor air quality, and avoiding exposure to strong odors.

Based on conventional care, the intervention group received EBN intervention. Specifically, the following steps were taken: First, an EBN team was established. Before taking up their positions, team members were required to undergo training and assessment to ensure they possessed strong work capabilities, a sense of responsibility, rich experience, and a comprehensive grasp of relevant knowledge. They also received regular training on the principles and concepts of evidence-based nursing and were involved in formulating nursing plans. Additionally, potential challenges that patients with MI undergoing PCI and confirmed postoperative reperfusion injury might encounter during the nursing intervention process were identified. Databases, journals, and professional websites were utilized to retrieve research findings related to these issues, including clinical studies, expert consensus, and conference records, while the credibility of the information was screened. The quality of the materials was assessed, with attention paid to the design rationale, sample size, feasibility of interventions, and the credibility of outcomes. Only rigorously evaluated materials were used as the basis for developing nursing plans. Meanwhile, nursing plans, where nursing staff were formulated; based on the retrieved findings and their own clinical observations of patients with MI undergoing PCI and experiencing reperfusion injury, along with the individual circumstances of the patients, nursing staff selected the most appropriate nursing plan. The nursing plans were first self-evaluated within the team. Subsequently, meetings were organized with healthcare professionals who had extensive clinical experience to evaluate the aforementioned plans, ensuring the rationality and effectiveness of the nursing content. This was then incorporated into the training to strengthen the team members’ understanding and was subsequently applied in clinical practice. Furthermore, the nursing plan was implemented; during this process, the patient’s responses were recorded to provide experimental data for subsequent research. In addition, the nursing outcomes were evaluated; the plan was optimized based on whether the expected results were achieved. The plan was also continuously improved; nursing staff constantly stayed updated on the latest nursing knowledge to enhance their professional competence and nursing standards.

### Observation indicators

We employed echocardiography to assess the cardiac function indicators, specifically the left ventricular ejection fraction (LVEF) and left ventricular end-diastolic diameter (LVEDD), before and after the intervention. LVEF was calculated using the formula: LVEF = (end-diastolic volume (EDV) - end-systolic volume (ESV))/EDV × 100%. The measurement of LVEDD was conducted utilizing the principle of ultrasound reflection to generate images of the heart. A professional physician acquired images of the left ventricle from the parasternal long-axis view and measured the LVEDD.

We collected 5 mL of fasting peripheral venous blood from patients in the morning to measure the levels of cardiac injury markers, including N-terminal pro-brain natriuretic peptide (NT-proBNP) and cardiac troponin I (cTnI). The measurements were performed using a Beckman Coulter immunoassay analyzer (model: Dxl 800/Access 2, purchased from Beckman Coulter), strictly adhering to the step-by-step instructions outlined in the user manual. The cut-off value for NT-proBNP is set at 300 pg/mL; the normal range for cTnI is 0–0.3 ng/mL.

To evaluate changes in patients’ psychological states before and after the intervention, we utilized the Self-Rating Anxiety Scale (SAS) and the Self-Rating Depression Scale (SDS). Higher scores on these scales indicated more pronounced levels of anxiety and depression in patients.

We monitored the incidence of complications, including ventricular fibrillation, vagal reflex, hypotension and acute thrombosis, over a 3-month follow-up period. During this time, we also recorded any cases of sudden death. Nursing satisfaction was assessed using a custom-designed questionnaire from our hospital, with a total of 20 items, each with five points, for a total of 100 points. A score of ≥80 points was highly satisfied, 60–80 points was satisfied, and <60 points was dissatisfied. The satisfaction rate was calculated as (highly satisfied + satisfied)/total cases × 100%. The pre-experimental exhibited a content validity of 0.73, and Cronbach’s α coefficient stood at 0.85.

### Statistical analysis

Statistical analysis was carried out using SPSS 22.2 and GraphPad 10.0. Data that followed a normal distribution were presented as mean ± standard deviation (x ± s). Independent sample t-tests were used for comparisons between groups, while paired sample t-tests were applied for comparisons before and after interventions. Categorical data were expressed as percentages (%), and the χ^2^ test or Fisher’s exact test was utilized for inter-group comparisons. A *P*-value of less than 0.05 was considered statistically significant.

## Results

### Baseline characteristics of the study subjects

Ninety eligible patients were randomly divided into two groups: the intervention group and the control group, with 45 cases in each group. The intervention group comprised 29 males and 16 females, aged 43–65 years, with an average age of 53.69 ± 6.65 years. The control group included 28 males and 17 females, aged 42–66 years, with an average age of 55.84 ± 6.60 years. No significant differences were observed in the baseline characteristics between the two groups (*P* > 0.05), ensuring the comparability of the study results.

### Levels of cardiac function indicators

**FIGURE 1:**
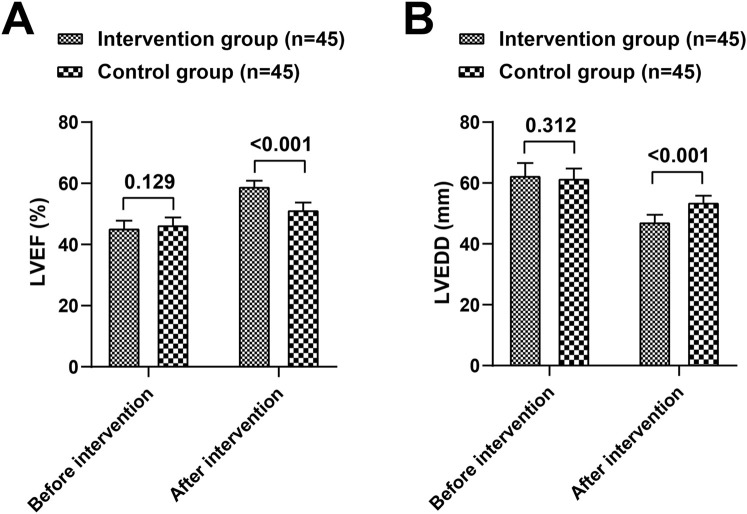
Comparison of cardiac function indices LVEF **(A)** and LVEDD **(B)** between the intervention and control groups.

Before the intervention, there were no significant differences in LVEF and LVEDD measurements between the two groups (*P* > 0.05). Following the intervention, both groups showed a significant increase in LVEF and a significant decrease in LVEDD compared to pre-intervention levels (*P* < 0.05). Additionally, the intervention group exhibited better LVEF and LVEDD outcomes than the control group (t = 12.550, 10.150, *P* < 0.05) (Table 1; Figure 1).

**TABLE 1:** Comparison of cardiac function indices LVEF and LVEDD between the intervention and control groups.

Group	n	LVEF (%)		LVEDD (mm)	
		Before	After	Before	After
Intervention group	45	45.53 ± 3.19	59.15 ± 2.62^*^	62.66 ± 4.87	47.36 ± 3.11^*^
Control group	45	46.56 ± 3.20	51.48 ± 3.16^*^	61.71 ± 3.97	53.83 ± 2.93^*^
t		1.533	12.550	1.018	10.150
*P*		0.129	<0.001	0.312	<0.001

Note: Compared with the pre-intervention values within the same group. *P < 0.05.

### Levels of myocardial injury markers

**FIGURE 2:**
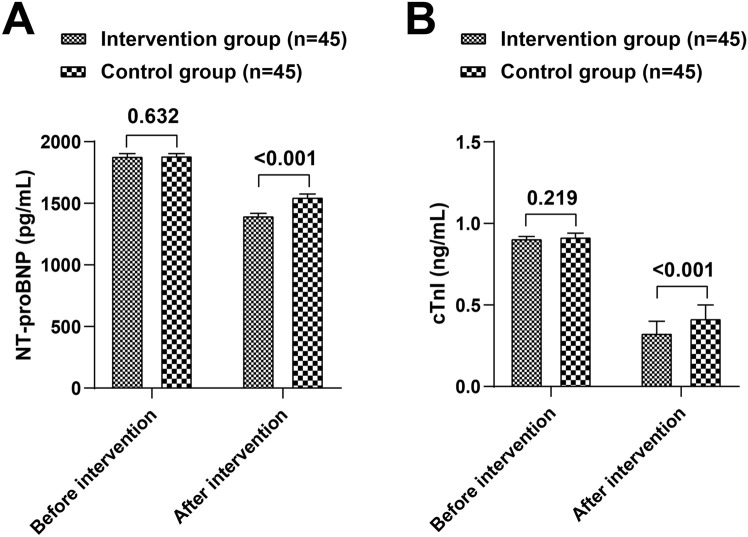
Comparison of myocardial injury marker NT-proBNP **(A)** and cTnI **(B)** between the intervention and control groups.

Prior to the intervention, no significant differences were observed in NT-proBNP and cTnI levels between the two groups (*P* > 0.05). Post-intervention, both groups demonstrated a significant reduction in NT-proBNP and cTnI levels compared to baseline (*P* < 0.05). Furthermore, the intervention group had lower NT-proBNP and cTnI levels than the control group (t = 16.396, 4.759, *P* < 0.05) (Table 2; Figure 2).

**TABLE 2:** Comparison of myocardial injury markers NT-proBNP and cTnI between the intervention and control groups.

Group	n	NT-proBNP (pg/mL)		cTnI (ng/mL)	
		Before	After	Before	After
Intervention group	45	1886.97 ± 43.08	1403.75 ± 41.62^*^	0.91 ± 0.03	0.33 ± 0.09^*^
Control group	45	1891.17 ± 39.60	1556.28 ± 46.50^*^	0.92 ± 0.04	0.42 ± 0.10^*^
t		0.481	16.396	1.238	4.759
*P*		0.632	<0.001	0.219	<0.001

Note: Compared with pre-intervention in the same group. *P < 0.05.

### Psychological status

**FIGURE 3:**
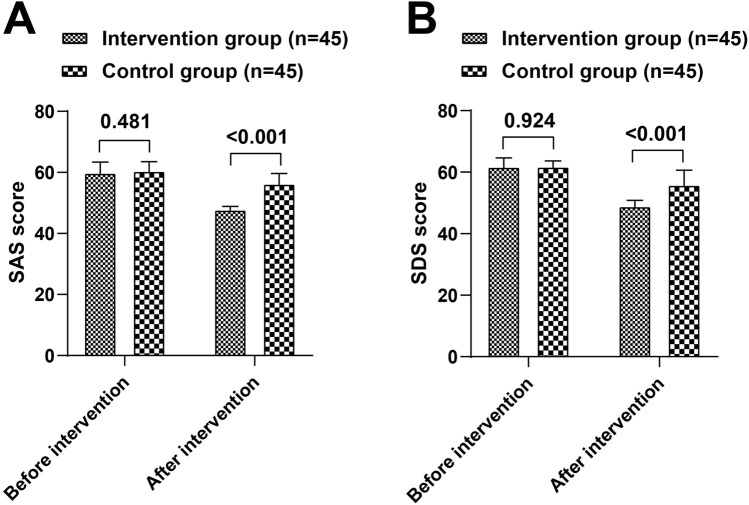
Comparison of SAS **(A)** and SDS **(B)** scores between the intervention and control groups.

Before the intervention, there were no significant differences in SAS and SDS scores between the two groups (*P* > 0.05). After the intervention, both groups showed a decrease in SAS and SDS scores compared to pre-intervention levels (*P* < 0.05). The intervention group had significantly lower SAS and SDS scores than the control group (t = 12.218, 7.336, *P* < 0.05) (Table 3; Figure 3).

**TABLE 3:** Comparison of SAS and SDS scores between the intervention and control groups.

Group	n	SAS		SDS	
		Before	After	Before	After
Intervention group	45	59.89 ± 4.43	47.80 ± 1.95^*^	61.76 ± 3.79	48.98 ± 2.78^*^
Control group	45	60.51 ± 3.90	56.31 ± 4.25^*^	61.82 ± 2.77	55.89 ± 5.67^*^
t		0.707	12.218	0.095	7.336
*P*		0.481	<0.001	0.924	<0.001

Note: Compared with pre-intervention within the same group. *P < 0.05.

### Complication rates and mortality

The intervention group had significantly lower rates of complications (χ^2^ = 5.120) and sudden death compared to the control group (*P* < 0.05) (Table 4).

**TABLE 4:** Comparison of complication rates and sudden death rates between the intervention and control groups.

Group	n	Acute thrombosis	Ventricular fibrillation	Vagal reflex	Hypotension	Incidence rate	Sudden death
Intervention group	45	0 (0)	1 (2.22)	1 (2.22)	1 (2.22)	3 (6.67)	0 (0)
Control group	45	3 (6.67)	4 (8.89)	2 (4.44)	3 (6.67)	12 (26.67)	6 (13.33)
χ^2^						5.120	-
*P*						0.021	0.026

### Satisfaction with nursing care

Patients in the intervention group reported significantly higher satisfaction levels with nursing care compared to those in the control group (*P* < 0.05) (Table 5).

**TABLE 5:** Comparison of patient satisfaction between the intervention and control groups.

Group	n	Highly satisfied	Satisfied	Dissatisfied	Satisfaction rate
Intervention group	45	31 (68.89)	14 (31.11)	0 (0)	45 (100.00)
Control group	45	25 (55.56)	12 (26.67)	8 (17.78)	37 (82.22)
*P*					0.006

## Discussion

Reperfusion injury is a complex inflammatory condition caused by factors such as lack of oxygen, metabolic stress, immune system activation, cell death processes, and the movement of white blood cells into tissues (Fernandez et al., 2020). Reperfusion injury following PCI in patients with MI remains a significant clinical challenge, often leading to worsened cardiac function, psychological distress, and increased morbidity and mortality (Mitsis and Gragnano, 2021). EBN interventions have emerged as a promising approach to mitigate these adverse outcomes by integrating the best available evidence with clinical expertise and patient preferences (Cardoso et al., 2021). This study evaluated the physiological and psychological effects of EBN in MI patients experiencing reperfusion injury post-PCI. The results demonstrated that EBN significantly improved cardiac function, reduced myocardial injury, alleviated psychological distress, and enhanced patient satisfaction compared to routine nursing care.

We found that after the nursing intervention, the intervention group had better results in LVEF and LVEDD, and lower levels of NT-proBNP and cTnI compared to the control group. As confirmed by the research results of Yanyun Zhou et al., after the implementation of EBN, patients’ LVEF increased, while left ventricular diastolic diameter and BNP decreased (Zhou et al., 2021). Xiaolan Liu et al.'s study also reports that EBN combined with exercise rehabilitation helps enhance myocardial contractility and improve cardiac function (Liu X. et al., 2022).

Moreover, the scores of the SAS and SDS in the intervention group were lower than those in the control group. This further supports the findings of Yanyun Zhou et al., that EBN can improve SAS and SDS scores and quality of life in patients with acute MI complicated by heart failure (Zhou et al., 2021). It is well-known that depression has a negative impact on the prognosis of coronary heart disease patients, including increasing the risk of recurrence and death (Pizzi et al., 2012). Studies have shown that depressive and anxious emotions can affect the function of the cardiovascular system through various pathways such as neuroendocrine and immune systems, thereby increasing the risk of cardiovascular events (Belvederi Murri et al., 2020; van Zutphen et al., 2023). The reduction in SAS and SDS scores in the intervention group in this study implies the alleviation of patients’ negative emotions, which may help reduce the risk of recurrence and death from cardiovascular diseases. In other words, clinical healthcare professionals should not only focus on the improvement of patients’ physiological indicators but also attach great importance to their psychological states and actively adopt effective psychological intervention measures.

Furthermore, the lower incidence of complications and mortality in the intervention group supports evidence that EBN can reduce adverse events and improve survival rates in high-risk patients (Zhang et al., 2022). Meanwhile, patients’ satisfaction with nursing care in the intervention group was higher than that in the control group. This further supports the report of Jun Meng et al. (Meng et al., 2022). This has important clinical implications, meaning that EBN can not only improve patients’ physiological and psychological indicators but also effectively reduce the risks they face. Moreover, EBN fully considers patients’ needs and feelings during the nursing process, emphasizing communication and interaction with patients, thus providing them with higher-quality and more humanized nursing services.

In light of the above, the application of EBN can be considered for different patient populations or healthcare facilities. Given the differences among patient groups, for elderly patients, taking into account their characteristics of multiple comorbidities and declining physical functions, personalized medication and nursing plans should be developed based on evidence, with physiological indicators such as heart and lung functions serving as references. Psychologically, personalized psychological interventions should be implemented according to psychological research to alleviate their anxiety and fear. For young patients, a progressive rehabilitation plan can be designed in conjunction with their occupational characteristics, incorporating occupational rehabilitation guidance. Online psychological support should also be provided, utilizing the internet to offer online communication and consultation services to relieve psychological pressure. At the healthcare facility level, large comprehensive hospitals can leverage advanced equipment and professional personnel to establish rehabilitation centers and form multidisciplinary teams to provide comprehensive EBN care. They can also consider conducting clinical research to explore better nursing models and undertake teaching tasks to cultivate EBN talents. Primary healthcare institutions, on the other hand, should focus on health education and extended nursing services. Meanwhile, they should establish a two-way referral mechanism with higher-level hospitals to enhance their nursing capabilities.

In conclusion, this study highlights the significant role of EBN in improving outcomes for MI patients with reperfusion injury post-PCI. By demonstrating improvements in cardiac function, psychological wellbeing, and patient satisfaction, it underscores the value of integrating evidence-based practices into routine nursing care. At the same time, this study still has some limitations that need to be improved. Firstly, some endpoint indicators, such as patient satisfaction, are highly subjective and may be influenced by various factors such as personal expectations and emotional states, which may lead to deviations in the research results. In addition, the study’s follow-up period was set at 3 months, which is relatively short for assessing the continuous changes in cardiac function and mental health, and failed to observe the long-term effects of EBN intervention on MI patients with reperfusion injury after PCI. Future studies can attempt to introduce more objective and quantifiable assessment indicators to reduce the impact of subjective factors on the research results. Meanwhile, extending the follow-up time to observe the long-term trends in patients’ cardiac function and mental health. Multi-center, large-sample studies can also be conducted to increase the representativeness of the research.

## Data Availability

The original contributions presented in the study are included in the article/supplementary material, further inquiries can be directed to the corresponding author.
